# Development of a liver graft assessment expert machine-learning system: when the artificial intelligence helps liver transplant surgeons

**DOI:** 10.3389/fsurg.2023.1048451

**Published:** 2023-09-22

**Authors:** Beatriz Pontes Balanza, Juan M. Castillo Tuñón, Daniel Mateos García, Javier Padillo Ruiz, José C. Riquelme Santos, José M. Álamo Martinez, Carmen Bernal Bellido, Gonzalo Suarez Artacho, Carmen Cepeda Franco, Miguel A. Gómez Bravo, Luis M. Marín Gómez

**Affiliations:** ^1^ Department of Computer Languages and Systems, Sevilla University, Seville, Spain; ^2^ HPB Surgery Unit, Virgen Macarena University Hospital, Seville, Spain; ^3^ HPB Surgery and Liver Transplant Unit, Virgen del Rocío University Hospital, Seville, Spain

**Keywords:** liver transplants, machine learning, decision-making process, liver graft assessment, artificial intelligence

## Abstract

**Background:**

The complex process of liver graft assessment is one point for improvement in liver transplantation. The main objective of this study is to develop a tool that supports the surgeon who is responsible for liver donation in the decision-making process whether to accept a graft or not using the initial variables available to it.

**Material and method:**

Liver graft samples candidate for liver transplantation after donor brain death were studied. All of them were evaluated “*in situ*” for transplantation, and those discarded after the “*in situ*” evaluation were considered as no transplantable liver grafts, while those grafts transplanted after “*in situ*” evaluation were considered as transplantable liver grafts. First, a single-center, retrospective and cohort study identifying the risk factors associated with the no transplantable group was performed. Then, a prediction model decision support system based on machine learning, and using a tree ensemble boosting classifier that is capable of helping to decide whether to accept or decline a donor liver graft, was developed.

**Results:**

A total of 350 liver grafts that were evaluated for liver transplantation were studied. Steatosis was the most frequent reason for classifying grafts as no transplantable, and the main risk factors identified in the univariant study were age, dyslipidemia, personal medical history, personal surgical history, bilirubinemia, and the result of previous liver ultrasound (*p* < 0.05). When studying the developed model, we observe that the best performance reordering in terms of accuracy corresponds to 76.29% with an area under the curve of 0.79. Furthermore, the model provides a classification together with a confidence index of reliability, for most cases in our data, with the probability of success in the prediction being above 0.85.

**Conclusion:**

The tool presented in this study obtains a high accuracy in predicting whether a liver graft will be transplanted or deemed non-transplantable based on the initial variables assigned to it. The inherent capacity for improvement in the system causes the rate of correct predictions to increase as new data are entered. Therefore, we believe it is a tool that can help optimize the graft pool for liver transplantation.

## Introduction

Since the last century, liver transplantation (LT) is the treatment of choice for both acute and chronic terminal hepatopathies (1, 2). Throughout these years, LT has evolved profoundly, presenting significant improvements in its short- and long-term survival results, for both grafts and recipients. This has led to a growth in the number of indications, increasing the number of patients on waiting lists for LT worldwide. This situation, together with the change in the profile of donors in recent decades, has created an imbalance between the number of candidates and the number of grafts available for LT (2–4). To increase the number of grafts and balance this situation, novel strategies for obtaining liver grafts have been developed: living donor, donor after cardiac death, split, domino transplant, and donors with expanded criteria (5). However, the strategies related to the process of liver graft assessment that enables reducing the rate of non-transplantable liver graft (NTLG), ruled out for LT that could have been transplanted, have yet to be explored. NTLG is the term used to describe liver grafts that are disposed of after an “*in situ*” evaluation, while transplantable liver graft (TLG) describes liver grafts that are transplanted after that.

In Spain, 13.1% of the offered liver grafts are discarded initially and 27.6% after an “*in situ*” assessment. This liver graft assessment is a complex process, and the most important tool that liver transplant surgeons (LTS) have, to carry it out, is their own experience in evaluating liver grafts for transplantation (3, 6). To do so, the LTS has the macroscopic features of the liver graft and the features of the liver donor collected in the liver donation protocol (LDP). A liver graft biopsy can help one decide whether the liver graft is transplantable or not. However, on many occasions, the biopsy cannot be performed or is inconclusive (7). Other complementary tests that can help the LTS in the decision-making process are imaging tests, which are good tools for studying future liver grafts and diagnosing absolute contraindications for transplantation (liver cirrhosis, cancers in any localization, several vascular atheromatosis, etc.). They can even provide very important information to the LTS before a laparotomy is performed (8). However, nowadays, the two main reasons for discarding a liver graft by the LTS are liver steatosis and the macroscopic aspect of the liver graft (9). Steatosis is hardly diagnosed by imaging tests (10), and the macroscopic aspect is an indeterminate reason given for NTLG that generates uncertainty since a real and concrete cause that justifies the liver graft discharge does not exist. In this context, imaging tests are not very useful. So, to reduce the NTLG rate that could have been transplanted, it is mandatory for development tools to provide additional information to the LTS about the liver graft that is being valued.

Recently, mathematical models based on artificial intelligence (AI) techniques have been developed in different medical fields, LT included (11–16). They relate to complex mathematical algorithms that, specifically in this area, demonstrate their utility in organ failure prediction and the donor–recipient pairing process of the liver graft (14–16). However, its contribution to the field of liver graft assessment has not yet been researched extensively. Nowadays, this situation is changing, and the interest of the scientific community in the role of AI in the liver graft assessment process is growing. Lately, a system based on this novel mathematical technology has been developed for estimating liver graft steatosis using the liver graft visual aspect with well-published results (10).

Machine learning (ML) is the area of AI that enables the creation of computer models with the ability to learn from real data. In this work, we use prediction systems that are trained from a set of labeled records and generate a model able to classify new data (17). There are innumerable applications of this technology in medical classification systems (18, 19). Specifically, in the LT field, ML has been used for screening and selecting LT recipients and predicting post-LT survival and complications (20). This novel technology can be used to predict the outcome of a new observation, based on a training data set with previous observations where the outcome is known. They can detect complex non-linear relationships between numerous variables that are used for predictive applications (21). So, a machine-learning algorithm, developed from the LTS with extensive experience, may be able to predict the liver graft suitability for LT, helping the LTS in the decision-making process and diminishing the NTLG rates that could have been transplanted.

The main objective of the study is to develop a tool that supports the surgeon responsible for liver donation in the decision-making process whether to accept a graft or not using the initial variables available to it.

## Material and method

### Data collection

The liver graft samples evaluated for LT between the period 2016 and 2018 were studied. They are composed of TLG and NTLG. All of them were evaluated by the same three LTS, which share the same validation criteria for liver graft assessment. The liver donor variables were extracted from LDP and collected prospectively in a database (Table 1). LDP is an official document that the coordination responsible for the liver offer has the obligation of designing and sending to the Organización Nacional de Trasplante (ONT). It contains the most important features of the liver donor, it is a common document for all Spanish liver transplantation groups, and its purpose is to facilitate the initial liver graft assessment by the surgical team responsible for LT (7).

**Table 1:** Descriptive study of the sample.

Variable	Qualitative variables	Continuous variables
Age		60.8 ± 14.1
Sex		
Male	(*n* = 190) 54.2%	
Female	(*n* = 160) 46.7%	
BMI		28 ± 4.9
Arterial hypertension	(*n* = 197) 56.2%	
DM	(*n* = 71) 20.8%	
DLP	(*n* = 118) 33.7%	
Personal	(*n* = 187) 53.4%	
Medical		
History		
Personal		
Surgical	(*n* = 75) 21.4%	
History		
GOT		47.4 ± 49.6
GPT		45.8 ± 189.6
GGT		76.7 ± 108.8
Bb T		0.6 ± 0.4
Na		146 ± 9.1
Amines	(277) 79.1%	
Amines dose		0.19 ± 0.23
Ultrasound	Normal (*n* = 257) 73.4% Pathological (*n* = 65) 18.5% Not performed (*n* = 28) 8.0%	
AcHBVc	(*n* = 35) 10%	
AcHCV	(*n* = 5) 1.4%	

AcHBVc, antibodies against hepatitis B capsid antigen; AcHBVc, antibodies against hepatitis C virus; AH, arterial hypertension; Bb T, bilirubinemia; BMI, body mass index; DLP, dyslipidemia; GGT, gamma-glutamil transferase; GPT, glutamate pyruvate transaminase; GOT, glutamic oxaloacetic transaminase; DM, diabetes mellitus; PMH, personal medical history; PSH, personal surgical history; Na, sodium.

Qualitative variables expressed in percentage (%), and continuous variables expressed in their own units: age in years; BMI in kg/m^2^; weight/height^2^ in kg/m^2^; GGT, GOT, and GPT in IU/ml; Na and Bb T in mg/ml; and dose of amines in μg/kg/min.

The reasons for rejections argued by the LTS were collected (Table 2). Just as LDP, it is an official document used by the LTS in which the reasons that can argue for classifying a liver graft as NTLG are collected, and it must be completed; it is made up of a closed list of reasons to discard the graft. Some of the reasons are indeterminate, and they are often the results of a macroscopic evaluation without histological confirmation.

**Table 2:** Reason given for NTLG.

Reason given for NTLG	*n* (%)
Steatosis	39 (31.7%)
Fibrosis	12 (9.7%)
Cirrhosis	2 (1.6%)
Bad perfusion	1 (0.8%)
Atheromatosis	2 (1.6%)
Macroscopic features	6 (4.8%)
Other	5 (4.1%)
Steatosis + ischemia	7 (5.6%)
Steatosis + atheromatosis	8 (6.5%)
Steatosis + fibrosis	2 (1.6%)
Steatosis + macroscopic features	5 (4.1%)
Steatosis + bad perfusion	1 (0.8%)
Atheromatosis + macroscopic features	8 (6.5%)
Steatosis + fibrosis + macroscopic features	1 (0.8%)
Fibrosis + cirrhosis	1 (0.8%)
Fibrosis + cirrhosis + macroscopic features	2 (1.6%)
Fibrosis + macroscopic features	1 (0.8%)
Steatosis + macroscopic features	1 (0.8%)
Steatosis + atheromatosis + macroscopic features	4 (3.2%)
Fibrosis + cirrhosis + macroscopic features + atheromatosis	1 (0.8%)
Fibrosis + macroscopic features + other	4 (3.2%)
Ischemia + macroscopic features	1 (0.8%)
Bad perfusion + atheromatosis	1 (0.8%)
Fibrosis + macroscopic features + atheromatosis + macroscopic features + surgical problem	1 (0.8%0
Steatosis + cirrhosis	5 (4.1%)
Ischemia + atheromatosis	1 (0.8%)
Fibrosis + atheromatosis + macroscopic features	1 (0.8%)

A liver biopsy was made for all grafts belonging to the NTLG group once the LTS classified the liver graft as NTLG, and all histological diagnoses were collected (Table 3).

**Table 3:** Histological findings.

Histological diagnosis	*n* (%)
Non pathological	25 (20.4%)
Pathological	98 (79.6%)
Steatosis >30%	49 (39.8%)
Fibrosis	21 (17.3%)
Cirrhosis	12 (9.7%)
Cholestasis	1 (0.8%)
Steatosis ≥ 30% + fibrosis	4 (3.2%)
Ischemia/necrosis	3 (2.4%)
Steatosis ≥ 30% + cholestasis	4 (3.2%)
Fibrosis + cholestasis	1 (0.8%)
Steatosis > 30% + ischemia/necrosis	2 (1.6%)
Fibrosis + ischemia/necrosis	1 (0.8%)

Findings of NTLG biopsy.

### Inclusion and exclusion criteria

Only the liver after donor brain death (DBD) was included in this study. All liver grafts were initially accepted by telephone based on the characteristics of the LDP and evaluated “*in situ*” by an expert surgeon in liver donation. We used the “Guide to Quality and Safety of Organs Transplantation” (22) to assess the liver graft.

All NTLGs were discharged without histological confirmation. The biopsies were made after the LTS classified the liver graft as NTLG, all of them being carried out by liver expert pathologists belonging to the same pathological anatomy unit that shares the same liver graft study protocol. Those that were considered NTLG after an intraoperative histological study were excluded. The TLG group is formed by liver grafts that were used for LT and did not develop primary non-function or early allograft dysfunction. All of them have post-reperfusion biopsy findings that were not pathological. Those TLG that were considered TLG after the intraoperative histological study were excluded.

### Definitions

NTLG: liver grafts that were accepted by telephone but failed the “*in situ*” LTS evaluation and were not used for liver transplantation.

TLG: liver grafts that were accepted by telephone and passed the “*in situ*” evaluation. All of them were used for liver transplantation. No liver in this group developed primary non-function or early allograft dysfunction, and all of them have a post-reperfusion biopsy without pathological findings.

Personal medical history (PMH): refers to any systemic pathology other than the cardiovascular risk factors.

Personal surgical history (PSH): refers to any previous abdominal surgery.

In the ultrasound item (Table 1), we considered it pathological if it presents compatible findings with steatosis hepatic, cirrhosis, fibrosis, or any morphological abnormality (15).

The item “macroscopic appearance” (Table 2) is indeterminate and does not correspond with any of the other six reasons.

In our center, during the study period, we considered pathological steatosis if it was >30% and macrovacuolar. Currently, the pairing policy has changed in our region, and the donor–recipient matching is allowed, so grafts with steatosis of up to 60% are accepted.

### Study groups

The sample was divided into two groups: TLG and NTLG.

### Ethics statement

This study was approved by the hospital's ethics committee. All data were fully anonymized before we accessed them.

### Statistical study

The development of the expert machine-learning system for liver graft assessment has two different phases:

(A) NTLG risk factors study

In Phase A, the NTLG risk factors were identified through a univariant study. For it, a single center, retrospective and cohort study based on a prospective database that analyzes a liver graft sample was performed (Table 4).

**Table 4:** Risk factors for rejection.

Variable	TLG % (*N *= 227)	TLG mean (*N *= 227)	NTLG % (*N *= 123)	NTLG mean (*N *= 123)	Univariant *p*	Univariant, OR (95% CI)
Age		58.1 ± 15.6		63.5 ± 13.1	***p *≤ 0.05**	**1.278 (1.189**–**2.343)**
Sex						
Male	128 (56.4%)		62 (50.4%)		N/S	
Female	99 (43.6%)		61 (49.6%)			
BMI		27.56 ± 5.56		28.50 ± 4.41	N/S	
Arterial hypertension	128 (56.4%)		69 (56.1%)		N/S	
DM	41 (18.1%)		30 (24.4%)		N/S	
DLP	66 (29.1%)		52 (42.3%)		***p *≤ 0.05**	**1.440 (1.087–1.907)**
Personal					***p *≤ 0.05**	**1.950 (1.416**–**2.684)**
Medical	102 (44.9%)		85 (69.1%)			
History						
Personal					***p* ≤ 0.05**	**1.578 (1.182**–**2.105)**
Surgical	38 (16.7%)		37 (30.1%)			
History						
GOT		45.26 ± 46.2		49.73 ± 53.0	N/S	
GPT		47.0 ± 133		44.76 ± 56.6	N/S	
GGT		66.4 ± 114.7		87.1 ± 103	N/S	
Bb T		0.57 ± 0.37		0.86 ± 0.56	***p* ≤ 0.05**	**1.254 (1.136−1.908)**
Na		145.62 ± 10.4		146.4 ± 7.9	N/S	
Amines	176 (77.5%)		101 (82.1%)		N/S	
Amines dose		0.17 ± 0.22		0.21 ± 0.24	N/S	
Ultrasound	Normal 187 (82.4%) Pathological 24 (10.6%) Not performed 16 (7.0%)		Normal 70 (56.9%) Pathological 41 (33.3%) Not performed 12 (9.8%)		***p *≤ 0.05**	**1.986 (1.178**–**2.457)**
AcHBVc	22 (9.7%)		13 (10.7%)		N/S	
AcHCV	2 (0.9%)		3 (2.5%)		N/S	

AcHBVc, antibodies against hepatitis B capsid antigen; AcHCV, antibodies against hepatitis C virus, Bb T, bilirubinemia; BMI, body mass index; DLP, dyslipidemia; GGT, gamma-glutamil transferase; GPT, glutamate pyruvate transaminase; GOT, glutamic oxaloacetic transaminase; DM, diabetes mellitus; Na, sodium.

Risk factor of not valid univariant study. Qualitative variables expressed in percentage (%), continuous variables expressed in their own units. Univariant study results expressed through *p*-value, OR, and CI.

*P* values and confident interval are highlighted in bold.

The normal distribution of the sample was determined using the Kolmogorov–Smirnov test. We compared them and studied whether there are statistically significant differences between both groups, establishing the odds ratio (OR) that each variable presented. For this univariant study, the Chi-square test has been used for qualitative variables and the Student's *t*-test for continuous variables. All those variables that presented statistical significance (*p* < 0.05) were included in the study. Based on the obtained results in the univariant study, we have designed a computer system that can help in the decision-making process to determine the suitability or not of a graft.

(B) Expert machine-learning system for liver graft assessment developed

The objective of the whole process is to obtain a mathematical model capable of predicting the suitability of a liver graft for LT from the diverse factors under study. To do this, a classic supervised learning workflow is applied, consisting of three different phases: (1) pre-processing and selection of the most relevant attributes, (2) conducting tests with different mathematical models that optimize a certain fitness, and (3) validation of results to choose the best model.

The first step consists of a preliminary pre-processing phase that includes feature selection (FS). This procedure often improves the prediction rate of the classifiers (23), by choosing the attributes presenting the greatest contrast or divergence for each value in the target attribute (class). In our case, an ANOVA *F*-test was applied, then selecting those attributes with the highest statistical value, a sign of a greater difference between the values of these attributes for transplantable and no transplantable grafts.

To conduct the second step, we have opted for classifiers based on decision tree ensembles. An ensemble is a model resulting from the collaboration between several individual classifiers, such that the final decision is the result of a consensus or vote. One of the most widely used prediction models in this category is the XGBoost (24) classifier. Furthermore, all machine-learning models need to adjust their configuration parameters by adapting to the data provided during the training phase.

Both Phases 1 and 2 have been performed using TPOT (https://epistasislab.github.io/tpot/). TPOT is a Python-automated machine learning (AutoML) (25). This tool performs an automatic search between different combinations of selection and classification strategies, providing the best setup, along with the necessary configuration parameters in each phase. For this purpose, it combines feature engineering, model selection, and hyperparameter optimization, selecting and tuning an ML algorithm for each stage. This whole process leads to the higher efficiency of the system, outperforming the models designed by hand. We have set TPOT with 200 generations, optimizing the area under curve (AUC) score, and a cross-validation value equaling 10. In summary, the obtained ML pipeline for our liver graft data set consists of the following steps:

(1) Add several synthetic features, using Bernoulli, Naïve Bayes, and XGBoost classifiers as estimators.
(2) Add two new features, corresponding to the number of zero and non-zero features for each sample.
(3) Perform Feature Selection, using ANOVA *F*-value between label/feature.
(4) Perform classification with XGBoost classifier.

The final step is validating the model. To avoid overfitting (the resulting model does not generalize to other data), different validation techniques may be used. In our case, both hold-out and cross-validation have been used. Cross-validation (CV) (26) is an iterative process that consists of splitting the data in two. In every iteration, one part will be used to train the model while the other part will be used to test it. The classification rate is computed as the average of all tests performed. We have validated our experiments with five different configurations. First, five and 10 folders have been considered for CV (5-CV and 10-CV). Then, we have considered all but one instance in each iteration, which is the leave one out cross -validation (LOOCV) approach. Finally, we have conducted two additional experiments named as train–test (TT), which performs a traditional train and test splitter or hold-out validation. It splits the data into two folders, containing 80% of the instances for training the model and 20% for testing it. TT (100%) represents the best possible evaluation, where the whole data set has been used both as a train and a test set. Table 5 shows the score results for all these types of validations. Furthermore, since our data had been previously randomly shuffled, we have performed validations for 100 different re-orderings, reporting the average scores. This process is similar to repeated k-fold cross-validation, with the difference that in our case, a random shuffling of the data is performed beforehand. In addition, this process is also performed in the TT validations (see Table 5).

**Table 5:** Scores average for 100 different re-orderings.

Validation	Accuracy	Precision	Recall	F1-score	AUC
TT (20%)	72.01%	0.63	0.51	0.56	0.75
5-CV	71.84%	0.62	0.51	0.56	0.75
10-CV	72.54%	0.64	0.51	0.56	0.76
LOOCV	73.73%	0.66	0.51	0.58	0.78
TT (100%)	82.87%	0.81	0.66	0.73	0.91

This table presents the average scores obtained for five different types of validation: train and test with a proportion of 0.2 (TT), fivefold and 10-fold cross-validations, LOOCV, and train and test with a proportion of 1.0. For each of these validation methods, the table reports the values of five evaluation measures.

External validation has been excluded in this work mainly due to the sample size. Instead, we have used five different types of validations (see Table 5), three of which correspond to cross-validation, under different numbers of subsets. In this context, cross-validation is a preferred choice when dealing with small sample sizes as it maximizes the data utilization, provides reliable performance estimates, assesses model generalization, and facilitates effective hyperparameter tuning. It helps mitigate the limitations imposed by limited data availability and allows for a more thorough evaluation of the performance of the model. In the field of medical applications, cross-validation has been frequently used and accepted as a validation strategy, as in these very recent works on cancer classification or COVID-19 diagnosis (27–29). Furthermore, Bhat et al. in their publication include numerous references to machine-learning applications in both pre- and post-LT settings in which cross-validation is used as the primary validation method.

In the following, a brief explanation of the scores in Table 5 is provided, where positive instances refer to NTLG:

• Accuracy: a fraction of all correctly classified instances.
• Precision: the proportion of true positives out of all positive predictions.
• Recall: the proportion of true positives out of all actual positive instances.
• F1-score: the harmonic mean of precision and recall.
• AUC: the measure of separability. It indicates the extent to which a model is capable of distinguishing between classes.

## Results

### Training sample

A total of 350 liver grafts that were evaluated for LT were studied: 123 NTLG vs. 227 TLG. The mean age was 60 years old, and most donors were males. The most frequent cardiovascular risk factor in the sample was arterial hypertension (AH), and more than 50% of the sample had associated medical antecedents other than the cardiovascular risk factors. The sample presented obesity Grade 1, and the analytical values were in the physiological range. The positive donors for antibodies against hepatitis B capsid antigen (AcHBV_c_) or antibodies against hepatitis C virus (AcHCV) were scarce.

The amines and their respective doses used for donor maintenance were collets (Table 1).

The most frequent isolated reason argued by the LTS for determining a liver graft as NTLG was steatosis. Regarding the combination of factors, the most frequent were steatosis + atheromatosis (Table 2). The most frequent histopathological finding was also steatosis (Table 3).

**Figure 1:**
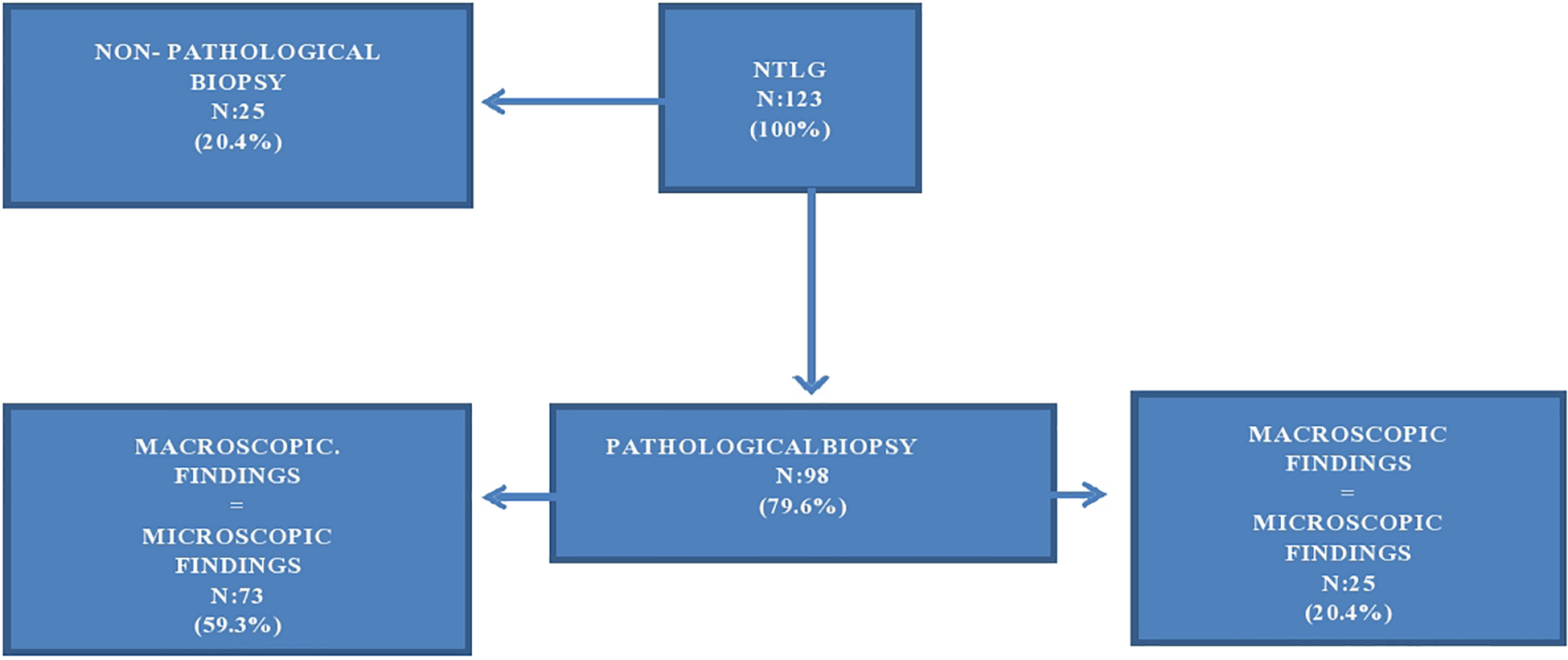
Biopsy findings of NTLG determinates by LTS. Flowchart.

In total, 79.6% of the NTLG had pathological biopsy results while the remaining 20.4% were reported without histopathological findings (Figure 1). In 59.3% of pathological biopsies, the reason argued by the LTS coincides with the histological diagnosis of the biopsy (concordance of 59.3%). In the remaining 20.3%, although the histological findings discarded the graft for liver transplant validity, they were different from those described macroscopically by the LTS (Figure 1).

### NTLG risk factors (univariant study results)

The NTLG risk factors identified in the univariant study were age, dyslipidemia (DLP), PMH, PSH, bilirubinemia (Bb T), and the result of previous liver ultrasound (*p* < 0.05).

The remaining 11 had no statistical significance (*p* > 0.05) (Table 4).

### Model results

**Figure 2:**
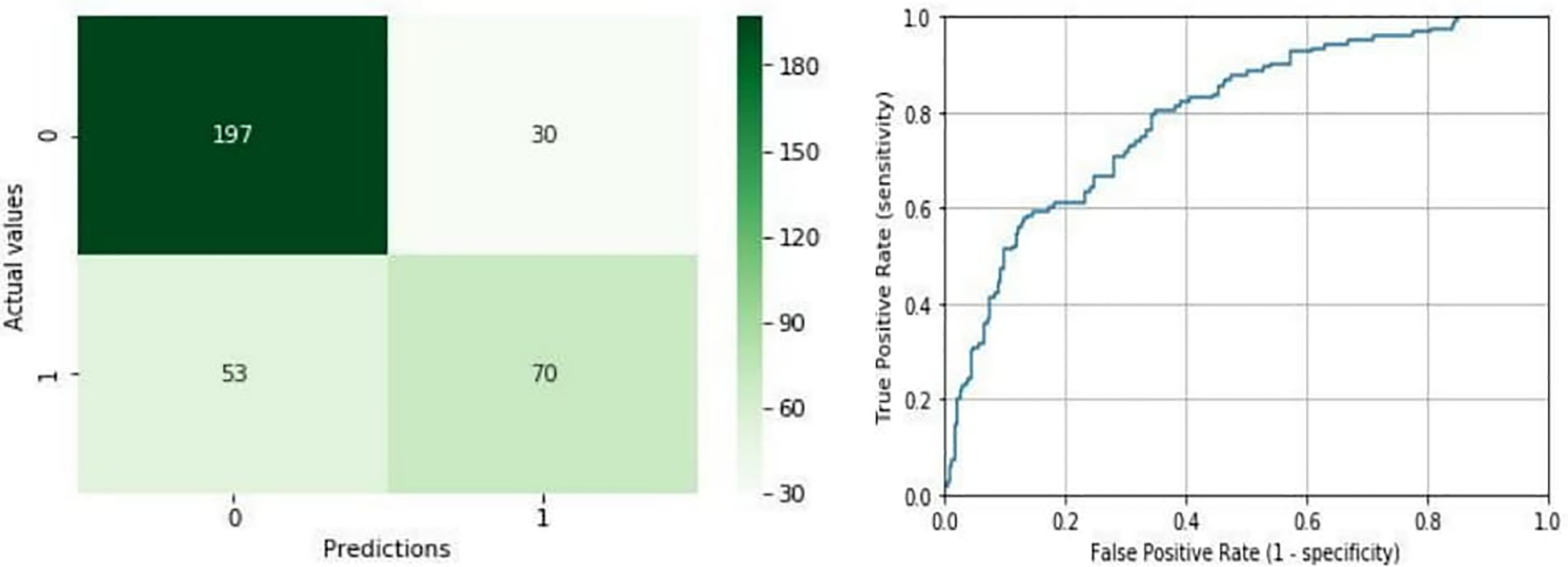
Confusion matrix and ROC curve for the best shuffle. Confusion matrix in the left shows the number of hits [true positives (TP) and true negatives (TN)] and errors [false positives (FP) and false negatives (FN)] for each class prediction. The ROC curve in the right represents the true positive rate (TPR) and false positive rate (FPR) of the model as the decision threshold of the classifier is varied. Its corresponding AUC is 0.79.

The best performance reordering in terms of accuracy and AUC corresponds to 76.29% of correct classifications, with an AUC value of 0.79 (precision = 0.7, recall = 0.57, F1-score = 0.63). Figure 2 (left) shows the number of hits and errors for each class value. In the first column of this matrix, it was observed that 197 out of 227 liver grafts classified as TLG for LTS were also classified as TLG for the mathematical model. Also, 53 liver grafts classified as NTLG for LTS were classified as TLG for the model, but 17 out of 53 liver grafts were reclassified as TLG for the mathematical model, belonging to the 25 liver grafts classified as NTLG for LTS with biopsy without pathological findings (Figure 1).

In the second column, 70 out of 123 liver grafts classified as NTLG for LTS were classified as NTLG for the mathematical model. In addition, 30 liver grafts classified as TLG for LTS were classified as NTLG for the model. As it can be derived from this matrix, the rate of success is considerably higher for TLG, which is 87% compared with 57% for NTLG. Figure 2 (right) represents the ROC curve corresponding to an area of 0.79.

In addition, a study of prediction reliability has been carried out. This is possible because the model provides the probability of confidence for each predicted label. Table 6 presents the hit probabilities for each interval of confidence, where the results have been divided into confidence intervals.

**Table 6:** Number of instances and hits by confidence intervals.

#instances	#hits	Hit probability	Interval of confidence	% of instances
42	29	0.690	0.5–0.59	12%
53	34	0.642	0.6–0.69	15.14%
67	44	0.657	0.7–0.79	19.14%
76	60	0.789	0.8–0.89	21.71%
112	100	0.893	0.9-	32%

This table shows the hits probabilities for five different intervals of confidence. Each row shows the number of predictions (#instances) falling within the specified interval, and the corresponding number of hits (#hits) and their probabilities. The percentage of the number of predictions (% of instances) for each interval of confidence is also included.

From Table 6, it can be derived that if the class is predicted with confidence between 0.5 and 0.6, the predictor is successful 29 times out of 42; however, if the confidence stands above 0.9, the prediction hit is almost 90%. The model not only provides a classification but also a confidence index of reliability. If the confidence provided is greater than 0.8 (more than half of the records), the probability that the prediction is successful is 160/188 = 0.85.

**Figure 3:**
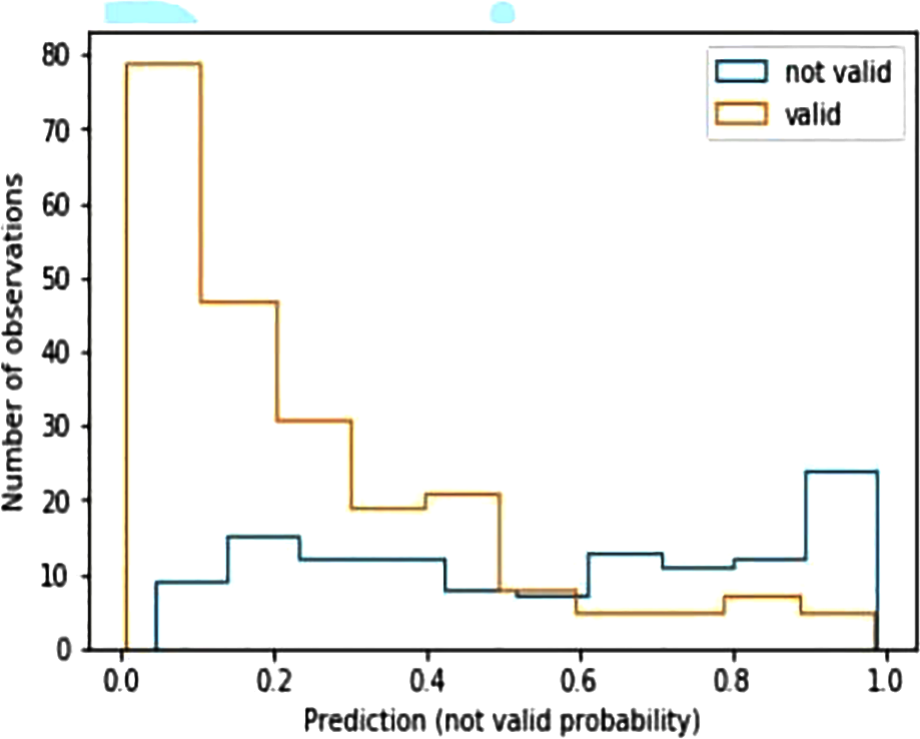
Histogram predictions of the best model. X-axis represents the probabilities of predictions for non-transplantable liver grafts. In the first half of the graph (x-axis), orange bars correspond to transplantable liver grafts correctly classified, while blue bars correspond to non-transplantable liver grafts misclassified. The second part of the graph represents the reverse situation.

The same distribution of hits and errors is depicted in Figure 3. In this chart, the probabilities of the prediction for the NTLG are represented on the x-axis. The bars in orange and blue represent the distribution of the valid and not valid instances, respectively. In the first half of the graph (x-axis), those TLG that have been correctly classified can be seen in orange, as well as those NTLG (in blue) that have been misclassified. Similarly, the second half of the chart shows in orange those TLG erroneously classified, as well as the NTLG (in blue) correctly classified. As can be seen, this chart confirms the previous numerical results, where the system shows much stronger behavior when predicting TLG than NTLG.

Furthermore, the XGBoost classifier provides us with a ranking specifying the relevance of variables. Specifically, the sorted list of variables in this study is the following: PMH, normal ultrasound, pathological ultrasound, PSH, Bb T, gamma-glutamil transferase (GGT), age, body mass index (BMI), DLP, amine doses, glutamate pyruvate transaminase (GPT), amines, AH, sodium (Na), and glutamic oxaloacetic transaminase (GOT). On the other hand, the remaining variables: sex, diabetes mellitus (DM), AcHCV, AcHBV, not performed ultrasound, have no contribution to determining graft validity, according to XGBoost.

In addition, we have conducted an overfitting analysis of our tool using learning curves. Learning curves provide insights into how the performance of the classifier evolves as the size of the training set increases. By plotting the training and validation accuracies of the classifier against the number of training instances, it is possible to identify if overfitting or underfitting is occurring.

**Figure 4:**
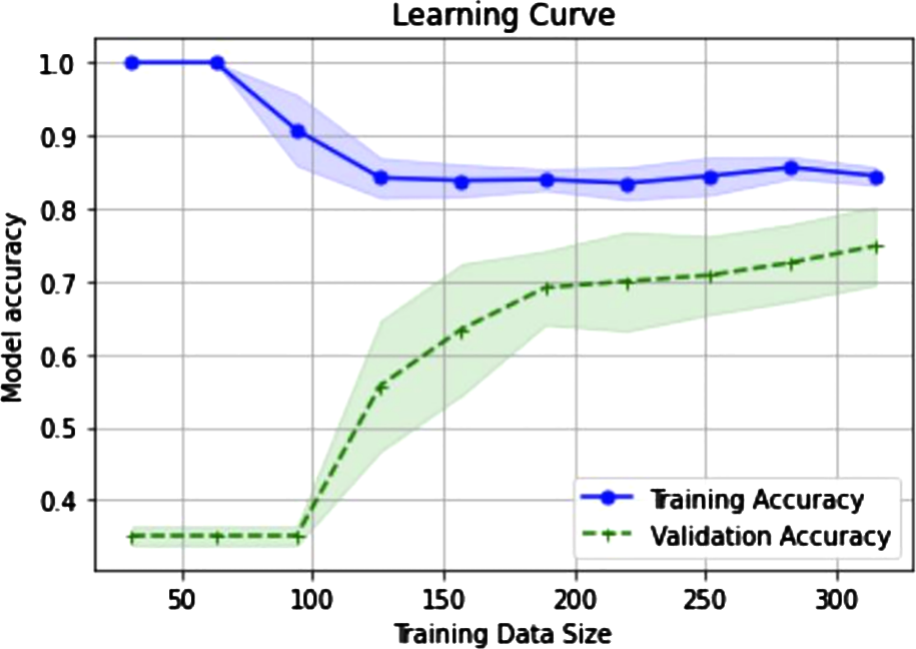
Learning curves to diagnose model performance. X-axis represents the incremental sizes of the training sets. Accuracy levels of both training and validation sets are represented in y-axis. It can be observed that both accuracies tend to converge as the size of the training sets increases.

In particular, the classifier is overfitting the training data when the training accuracy is high, and the validation accuracy remains significantly lower along the graph. This means that the classifier is memorizing the training examples and performing poorly on unseen data. On the other hand, when the training and validation accuracy curves converge at a certain accuracy level, it suggests that the performance of the classifier stabilizes, and it is not significantly affected by adding more training examples. The accuracy level of the convergence indicates a situation of underfitting (low level) or a well-generalized classifier (high level). As it can be seen in Figure 4, the convergence of both the training and validation curves of our tool occurs at a desired level of accuracy, indicating that our system generalizes well to unseen data.

## Discussion

In this study, we present a mathematical model that initially classifies the liver graft as transplantable or not based only on LDP information. LDP is an official ONT document that is sent to the liver transplant team that is valuing the liver graft, containing the most important features of the donor. It is the first information known and constitutes a very important element in the decision-making process. The information that the mathematical model gives us relates to the possibility of using the liver graft for LT. It does not match the donor–recipient, does not calculate the index of risk of primary graft failure (PGF), and does not make predictions about patient post-transplant mortality, survival, or complications.

The liver graft assessment for transplantation is a complex process. The two main tools that the LTS can use to carry out it are the LDP and the experience of the surgeon (30). This experience is not subjective; it is the result of the accumulated experience of each surgeon and is built on knowledge acquired for years by themselves, which improves that assessment capacity, increasing the number of correct evaluations. An important characteristic of this tool is that it has been designed based on the accumulated personal experience of not only one LTS but all the LTS of our Unit. Only a correct liver graft assessment can determine the optimal use of the current donor pool, avoiding the loss of potentially useful grafts. We aim to develop a tool that supports the surgeon responsible for liver donation in the decision-making process whether to accept a graft or not for LT, using the initial variables available to it.

Currently, the process for liver graft assessment can be improved; the rate of NTLG without pathological findings that confirm it is between 20.4%–35.5% (31, 32). It refers to liver grafts rejected for LT that could have been used for that. In addition, the two main reasons argued by the LTS for rejecting the liver graft are steatosis and macroscopic aspect; in Spain, these arguments constituted 21%–29.4% and 29.6%–31.5%, respectively, in the last 2 years (9, 33). Steatosis and the macroscopic graft features are two arguments that should be used with great caution. Both depend on a macroscopic valuation made by the LTS, which is based on their personal experience. For classifying a liver graft as NTLG for steatosis, the macroscopic and visual assessment is not enough, because it is not dependent on steatosis “*per se*,” but on the percentage and type of steatosis. On the other hand, the macroscopic features of the graft constitute an indeterminate item defined by the absence of the specific macroscopic criteria for rejection. In our study, up to 66.6% of the organs discarded for this reason presented a biopsy without pathological findings. In these cases, an intraoperative biopsy could help the surgeon make decisions, but it is not always possible to perform it.

For all that, there exists a percentage of liver grafts rejected for LT that could have been used for this purpose or that could have benefited from a more comprehensive assessment process, at least, based on the following:

• The argument of the surgeon: up to 36.5% of the grafts in our sample represent the total percentage of grafts discarded because of steatosis and macroscopic features. In the case of the national series in recent years, this percentage has ranged between 50.6% and 60.9% (9, 33).
• The biopsy reports: up to 60.2% in our series, the summation of NTLG with biopsy, without pathological findings and with steatosis findings.

The model proposed in this study can be a useful support tool in the process of liver graft assessment. It not only produces a prediction but also informs of the probability with which each prediction has been selected. The best results obtained by the system in terms of precision corresponded to 76.29% with an AUC value of 0.79. This success rate is established for a confidence interval of >0.8, with an error probability of 0.15. To design the model under the most realistic conditions possible, the inclusion criteria used were the LTS assessments and not the biopsy findings, since it is known later. Therefore, the system has been tested with data in which the group of grafts considered as NTLG contains grafts that could have been used (20.4%). Of these 25 NTLG that presented a biopsy without pathological findings, 17 belong to the 53 NTLG predicted by the mathematical model as a TLG. It is an interesting finding because it means that the model predicts TLG more accurately than NTLG, identifying TLG classified as NTLG by LTS, which is in this case, 17 grafts of 25 (68%). However, it is important to note that classifiers should not replace clinical expertise and domain knowledge. Instead, they should complement the decision-making process by providing additional insights and supporting doctors in their clinical judgment. In this context, using classifiers for making predictions in health applications offers several advantages over the traditional statistical techniques. On the one hand, it allows handling more complex relationships and interactions between multiple variables in the data, since they are well-suited to capture non-linear relationships. Also, classifiers can adapt and update their predictions as new data become available. This adaptability to changing data allows incorporating new evidence to the model, making them very suitable to real-time decision support.

The scientific community has already used math for aiding this complex process and make the liver graft pool available for LT profitable. Based on classical statistics, there are tools that calculate the index of risk of FPI, or even, the survival of the patient with crossed data of donors and recipients (34, 35). In addition, over the last few years, the number of studies researching to explore AI applicability in the LT field has been growing. The main areas of LT where this technology is being developed are screening and matching donor–recipient and post-transplant mortality, survival, and complication predictions (16, 20). In 2020, a very interesting study was published by Moccia et al. (10) using AI for determining liver graft steatosis. To do so, liver graft visual features are studied by using a smartphone. The authors propose a computer-assisted assessment that evaluates hepatic steatosis based on the visual characteristics of the graft. This is a very interesting approach that helps the LTS in the decision-making process. By going through two phases of training, supervised and semi-supervised, they develop a system capable of determining hepatic steatosis. There are two main differences with the model we propose:

• The assessment we perform with our model is global, based on donor characteristics. We do not focus on just one of the reasons of graft rejection.
• We do not need to travel to obtain the images to carry out the assessment. The ONT sends the LPD before moving the surgical team, so the assessment is performed very early. This avoids unnecessary travel, with the consequent economic savings for the health system.

As we previously described, we aim to develop a tool that supports the LTS in the decision-making process whether to accept a graft or not using the initial variables available to it. To make the most of the previous information on liver grafts provided by LDP, we propose to study and learn from the previous and accumulated experience of the LTS, using AI techniques. The LTS experience is based on a non-analytical model of clinical reasoning. The outcome of the decisions we make can be optimized by the analytical processes of our AI-based tool (36, 37). It is interesting, as doctors use hypothetico-deductive reasoning to arrive at a diagnosis. In this case, our system is based on the knowledge of previous experiences, which come from expert LTS, learning from their successes and mistakes. Therefore, it does not perform deductive reasoning but is based on the result of previous experiences. Thanks to it, the system manages to improve its future assessments as we introduce more information from previous assessments. To improve its predictions, the system requires new information, from which it learns. In this way, it improves its predictions. So, the system learns from accumulated experience.

AI can be understood as the part of science in charge of developing a set of mathematical algorithms that allow computers to perform processes such that they can pose solutions to determinate problems after reaching their conclusions, which are the result of analyzing a large amount of data (38). For a long time, this concept belonged to the field of science fiction; however, at present, it is a field with great growth and capacity for expansion that demonstrates its usefulness and applicability in different fields of science and, of course, in surgery. AI encompasses different areas of knowledge, and for this project, we have utilized ML. ML enables computers to identify patterns and make predictions based on them. In contrast to classical computer science programming, which relies on predetermined patterns (e.g., pressing a button always opens the same screen), ML allows for predictions without explicit programming by utilizing labeled data (supervised learning) (39, 40). By leveraging the provided labels, the computer learns to recognize patterns and make predictions accordingly. In this context, ML permits the computer to learn from its own mistakes when performing a certain task (41), leading to gradual improvement with each repetition of the function it was programmed for. In supervised learning, the algorithm is trained on a labeled data set, adjusting its internal parameters or model representation to minimize the difference between its predicted outputs and the true labels. This iterative learning allows the algorithm to refine its performance over time. In our specific case, XGBoost gradually improves its performance and predictive capability by making predictions on the validity or otherwise of transplant candidate liver grafts. The debate on the ability of machines to reason has been one of the most relevant topics in the field of AI. While it is true that advances in this area have enabled machines to perform increasingly complex tasks, such as voice recognition and machine translation, the consensus is that these machines do not reason in the human sense of the term. Instead, they learn patterns from data and build computational models based on those patterns. In the context of AI, ML is the most widely used approach to create systems capable of recognizing complex patterns in large data sets. ML is based on the idea that machines can learn from examples and then use that knowledge to make predictions about new data. However, this approach does not necessarily imply that machines reason in the same way that humans do, since human reasoning implies an ability to make inferences and use prior knowledge effectively (36, 42).

The main limitation of the development of this technology is the lack of data. AI needs large databases to serve as a source of information to develop new models of data analysis and interpretation and to improve the existing ones (39). For this, the collaboration between different surgical teams from different cities, communities, or even countries is very important. The development of national networks for information flow between the different surgical groups that allow for real and rapid updating of data is fundamental if we want to optimize and implement this technology in the 21st-century surgery (43).

Currently, we are in the initial development phase of this system.

The main limitations associated with our study are that it is a retrospective study; the mathematical model has been trained with the results of only one liver transplant team and must be externally validated. In addition, the tool has been trained with a pool of grafts in which steatosis of >30% was considered a criterion for graft rejection. The pairing policy has changed in our region, and donor–recipient matching is allowed, so grafts with steatosis of up to 60% are accepted. Therefore, the tool may classify grafts as NTLG that we can currently use. We hope that all these limitations will be addressed in the future as we train the tool, as machine learning can learn as it is trained with new data. Thanks to this capability, it can adapt to such modifications.

For all these reasons, we currently continue to introduce new data in the model, so we hope that the system improves its predictive capacity.

## Conclusions

The tool presented in this paper obtains a high accuracy in predicting whether a liver graft will be transplanted or deemed non-transplantable based on the initial variables assigned to it; thus, we think that it can become an important tool to help in the decision-making process supporting the valuation of the LTS. The fact that the model presents better results identifying TLG is very interesting because the final objective is to decrease the number of TLG discharged to liver transplantation that could have been used for LT.

AI and big data technology need the collaboration of the surgical community to advance. So, these systems learn and improve as they analyze more data. In this way, the creation of large databases that include as many patients as possible is necessary if we want to implement these novel mathematical technologies.

## Data Availability

The raw data supporting the conclusions of this article will be made available by the authors, without undue reservation.
